# Fluorine-18 Fluorodeoxyglucose PET/CT Images of Hidradenitis Suppurativa Mimicking Metastasis in a Patient With Small Cell Lung Carcinoma

**DOI:** 10.7759/cureus.11770

**Published:** 2020-11-29

**Authors:** Ozgul Ekmekcioglu, Kerim Sonmezoglu

**Affiliations:** 1 Nuclear Medicine, Sisli Etfal Education and Research Hospital, Istanbul, TUR; 2 Nuclear Medicine, Cerrahpasa Medical Faculty, Istanbul, TUR

**Keywords:** pet/ct, hidradenitis suppurativa, fdg, lung cancer

## Abstract

A 54-year-old male patient with metastatic small cell lung carcinoma received chemotherapy and in follow-up he was referred to positron emission tomography (PET)/CT scan for re-staging. In addition to the primary disease, increased Fluorine-18 fluorodeoxyglucose (F-18 FDG) uptake was detected in skin and subcutaneous fat tissue in the axillary, periscrotal, and perianal regions, which was later proven as hidradenitis suppurativa in PET/CT images.

## Introduction

Hidradenitis suppurativa is a chronic inflammatory disease of the apocrine glands caused by follicular occlusion in the epithelium and presents with recurrent abscess or sinus tract. The etiology of the disease is still unknown, and it is more common in women and not usually seen before puberty or after 40 years of age [1,2]. It is most commonly detected in axillary, inguinal, and perineal regions, but also can be seen in perianal, interior of thighs, inframammary fold, and genital areas [1-3]. It can cause scarring, abscess, fistula, and even squamous cell carcinoma has been shown in chronic disease [4,5]. It is not easy to determine the extensiveness of the disease. CT, MRI, and ultrasonography (USG) are already used for imaging hidradenitis suppurativa [5,6]. Since increased Fluorine-18 fluorodeoxyglucose (F-18 FDG) uptake is also detected in inflammatory tissue, it might not be easy to differentiate infectious disease from malignancy in PET/CT images [7,8]. To our knowledge there have not been many reports presented about PET/CT imaging of hidradenitis suppurativa [6,8,9]. Our aim is to present a patient with small cell lung cancer assigned for a PET/CT scan, who has also been detected for suspicious lesions for metastasis that later proven to be hidradenitis suppurativa.

## Case presentation

**Figure 1:**
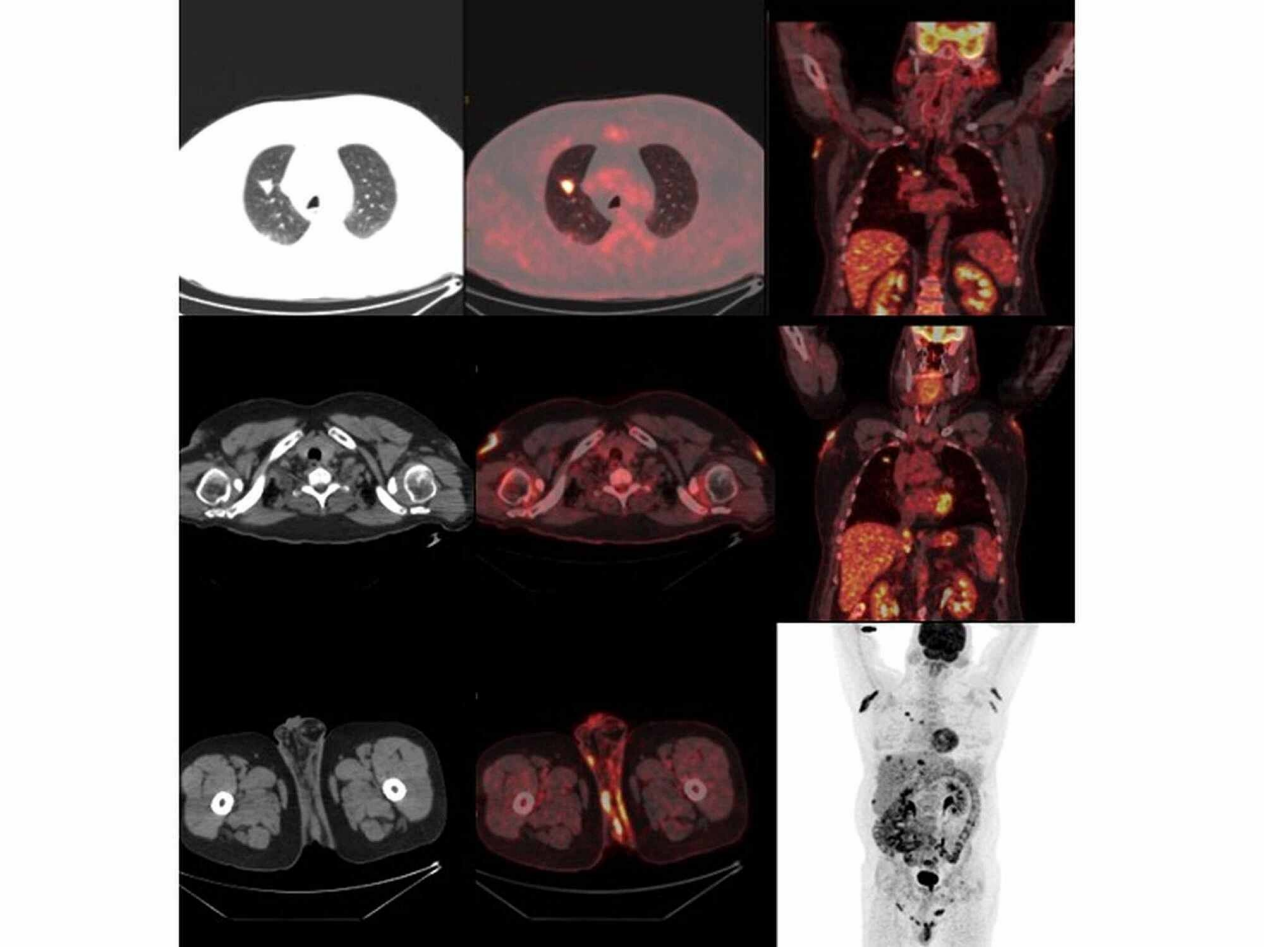
Primary tumor in lung and lesions in axillary and perianal region with increased FDG uptake

A 54-year-old male patient with small cell lung carcinoma, who had liver metastases and received chemotherapy, was referred to PET/CT scan for re-staging. He was in follow-up and had no previous PET scan, known clinical history, or co-morbidities. One hour after FDG injection PET/CT images were obtained from the whole body. A primary lesion with intense uptake was detected in the right upper lobe of the lung and lymphadenopathy with increased FDG uptake in the right paratracheal lymphatic region in the mediastinum. Additionally, multiple focal FDG uptake was detected in the liver parenchyma, which was compatible with known hepatic metastasis. Furthermore, increased FDG uptake was detected in skin and subcutaneous fat tissue in both axillary, periscrotal, and perianal regions suspicious for metastasic disease (Figure 1). 

**Figure 2:**
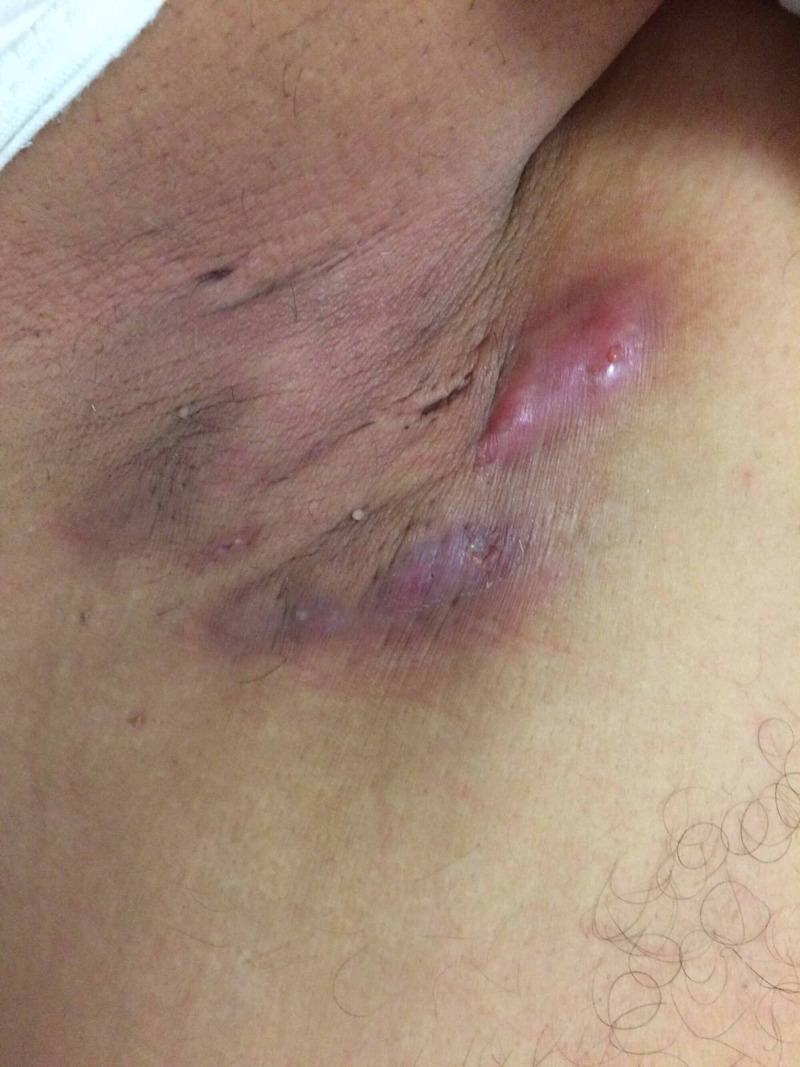
Erythematous skin lesions in axillary region

Patient was questioned about the skin lesions after the scan was performed. On physical examination, erythematous nodular lesions were detected especially in the axillary region with serous and purulent discharge (Figure 2). Biopsy was done subsequently and the histopathology report was compatible with chronic inflammation. On clinical examination, the patient had hidradenitis suppurativa.

## Discussion

Aside from malignancy, pathological FDG uptake can be detected in inflammatory tissue or any organs affected by infectious disease [7]. Inflammatory skin lesions have also been demonstrated to show increased FDG uptake [10]. 

Hidradenitis suppurativa is a skin condition of inflammatory reaction to follicular occlusion. It could present as nodular lesions or abscess formations [1,4]. It has been reported to mimic metastatic skin lesions such as lymphoma, malign melanoma, and lung carcinoma like in our case [11,12]. 

Cutaneous or subcutaneous lesions could be a sign of metastases from several cancer types like breast cancer, melanoma, or lung cancer [13,14]. The appearance of metastatic skin lesions may differ and present like an inflammatory lesion [15]. It has been reported that metastatic skin lesions spread either via lymphatic or hematogenous route. It is not very common to detect skin metastases in patients with small cell lung cancer, however when it presents a poorer prognosis is expected [16-18]. Consequently, histopathological confirmation with biopsy should be performed for suspicious skin or subcutaneous lesions to distinguish metastatic disease. 

## Conclusions

F-18 FDG PET/CT is useful for detecting primary tumors or metastases that usually presented with increased FDG uptake. Pathological FDG uptake could be also detected in infectious disease. Hence, it will be helpful for clinical and pathological evaluation to differentiate inflammatory tissue from malignancy when there is an unexpected FDG uptake on skin and subcutaneous tissue like in our case.
